# Interoception, cardiac health, and heart failure: The potential for artificial intelligence (AI)—driven diagnosis and treatment

**DOI:** 10.14814/phy2.70146

**Published:** 2025-01-09

**Authors:** Mahavir Singh, Anmol Babbarwal, Sathnur Pushpakumar, Suresh C. Tyagi

**Affiliations:** ^1^ Department of Physiology, School of Medicine University of Louisville Louisville Kentucky USA; ^2^ Center for Predictive Medicine (CPM) for Biodefense and Emerging Infectious Diseases School of Medicine, University of Louisville Louisville Kentucky USA; ^3^ Department of Epidemiology and Population Health, School of Public Health and Information Sciences (SPHIS) University of Louisville Louisville Kentucky USA

**Keywords:** automation, cardiovascular medicine, machine learning

## Abstract

“I see, I forget, I read aloud, I remember, and when I do read purposefully by writing it, I do not forget it.” This phenomenon is known as “interoception” and refers to the sensing and interpretation of internal body signals, allowing the brain to communicate with various body systems. Dysfunction in interoception is associated with cardiovascular disorders. We delve into the concept of interoception and its impact on heart failure (HF) by reviewing and exploring neural mechanisms underlying interoceptive processing. Furthermore, we review the potential of artificial intelligence (AI) in diagnosis, biomarker development, and HF treatment. In the context of HF, AI algorithms can analyze and interpret complex interoceptive data, providing valuable insights for diagnosis and treatment. These algorithms can identify patterns of disease markers that can contribute to early detection and diagnosis, enabling timely intervention and improved outcomes. These biomarkers hold significant potential in improving the precision/efficacy of HF. Additionally, AI‐powered technologies offer promising avenues for treatment. By leveraging patient data, AI can personalize therapeutic interventions. AI‐driven technologies such as remote monitoring devices and wearable sensors enable the monitoring of patients' health. By harnessing the power of AI, we should aim to advance the diagnosis and treatment strategies for HF. This review explores the potential of AI in diagnosing, developing biomarkers, and managing HF.

## Graphical abstract

Artificial intelligence (AI) and the human brain: The brain governs interoception by perceiving internal bodily sensations. AI advancements present valuable opportunities to deepen our understanding of this intricate process.

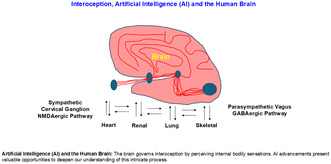

## 1 INTRODUCTION

Interoception is a term used to describe the process by which an organism perceives, interprets, and integrates internal physiological signals originating from within the body. These signals convey information about various aspects of bodily functions, such as heart rate, respiration, blood pressure, hunger, thirst, temperature, and visceral sensations. The awareness and interpretation of these internal bodily signals play a crucial role in regulating homeostasis, emotional experience, and self‐awareness. Further, interoception is closely related to the autonomic nervous system (ANS), which controls involuntary bodily functions. The ANS consists of two main branches: the sympathetic nervous system (SNS) and the parasympathetic nervous system (PNS). These systems work in harmony to maintain the body's internal balance, and interoception helps to monitor and regulate these processes. Interestingly, the interoceptive signals are conveyed by a variety of receptors, pathways, and macrovesicles/exosomes including those in the viscera (i.e., internal organs), blood vessels, and muscles. These signals are transmitted to the brain, where they are processed and integrated in regions like the insular cortex, anterior cingulate cortex, and other brain areas. The integration of interoceptive information is crucial for maintaining physiological stability and for generating emotional and motivational responses. In fact, interoception is a complex and multifaceted concept, and its study has gained increasing attention in various scientific fields, including psychology, neuroscience, and medicine. Researchers have explored the role of interoception in various contexts, including emotion regulation, decision‐making, self‐awareness, and mental health. Dysfunctions in interoceptive processing have been linked to various conditions, such as anxiety disorders, depression, eating disorders, and autism spectrum disorders (Barrett & Simmons, 2015; Craig, 2002; Khalsa & Lapidus, 2016; Seth, 2013; Seth & Tsakiris, 2018).

Again, interoception, the fundamental process of sensing and interpreting internal bodily signals, is crucial for maintaining physiological and psychological well‐being (Iodice et al., 2019). In fact, it is primarily linked to brain communicating with other organs; however, studies have also shown that during deep sleep (when brain is resting), the leg movements cause changes in heart rate, blood pressure and frequency in heart rate variability, suggesting that the skeletal is communicating with the heart. This means that in addition to brain, there is also parallel communication between the systemic organs (i.e., interoception by other organs along with the brain) (Malkiewicz et al., 2024). Further, in a self‐reported (interoceptive sensibility) and behavioral (interoceptive accuracy) study has suggested interoception phenomenon in elite (top 100 ranking) sprint and long‐distance runners, versus non‐athletes (Seabury et al., 2023). Thus, it could be assumed that any dysfunction in interoception may result in profound implications for both mental and cardiovascular health (Khalsa et al., 2018).

We explore the intricate relationship between interoception, mental health, and cardiovascular health, highlight the negative consequences of interoceptive dysfunction and its potential contribution to heart failure (HF) (Tsakiris & Critchley, 2016). We opine that impaired interoception disrupts the accurate perception and interpretation of internal signals, leading to difficulties in recognizing and understanding emotional states (Nord & Garfinkel, 2022). Such disruptions can potentially contribute to maladaptive emotional regulation and impact overall psychological well‐being. For example, mental health conditions such as anxiety, depression, and post‐traumatic stress disorder (PTSD), may be associated with interoceptive dysfunction, which can directly affect the cardiovascular health (Laricchiuta et al., 2023; Musey Jr. et al., 2020; Yoris et al., 2020). In fact, there are indications that interoceptive impairment are linked to cardiovascular disorders and an increased risk of HF. Because disruptions in interoception can disturb ANS regulation, resulting in imbalances in the cardiovascular response to potential stressors, these factors can manifest into increased heart rate, elevated blood pressure, and altered cardiac contractility, all associated with adverse cardiovascular outcome (Bonete et al., 2023). Additionally, impaired interoception can hamper the recognition of early signs of cardiovascular distress, potentially delaying timely medical intervention and exacerbating the progression of HF. Thus, understanding the intricate interplay between interoception, mental health, and cardiovascular health is crucial for developing comprehensive strategies to prevent, diagnose, and treat HF (Yoris et al., 2020).

By elucidating the underlying mechanisms of interoceptive dysfunction and its impact on mental and cardiovascular well‐being, we can identify novel intervention targets and develop personalized approaches to address both aspects of health. Further, advancements in technology, including the utilization of big data sets, artificial intelligence (AI) technology, machine learning (ML) tools, automation, electrocardiography (ECG) imaging, sonography images, and blood biomarkers, offer promising avenues for detecting and managing HF (Grün et al., 2020; Kwon et al., 2021; Li et al., 2022). AI a term that was coined by emeritus Stanford Professor John McCarthy in 1955. He defined AI as “the science and engineering of making intelligent machines” via simulation of human intelligence processes by machines, particularly computer systems. These processes include learning (the acquisition of information and rules for using it), reasoning (using rules to reach approximate or definite conclusions), and self‐correction. In fact, the AI applications encompass a broad spectrum, ranging from simple tasks like automation and data analysis to complex endeavors like natural language processing and autonomous decision‐making. AI systems can be categorized into two types: narrow AI, which is designed for a specific task, and general AI, which aims to mimic human cognition across a wide range of tasks. The field of AI continues to evolve rapidly, driven by advancements in ML, neural networks, and big data processing, with profound implications for various industries, including healthcare, finance, and transportation. In short, AI is a technology that enables computers and machines to simulate human intelligence and problem‐solving capabilities (Meskó & Görög, 2020; Noorbakhsh‐Sabet et al., 2019; Rajpurkar et al., 2022; Xu et al., 2021). ML is an evolving branch of computational algorithms that focuses on using the data, statistical models to analyze and draw inferences from that data to enable AI to imitate the way that humans learn thus gradually improving its accuracy from the patterns in the data. In simple words ML is a form of AI that can emulate human intelligence by learning from the surrounding data environment. Techniques and tools based on ML have been applied successfully in diverse fields ranging from pattern recognition, computer vision, spacecraft engineering, finance, entertainment, and computational biology to biomedical and medical applications. In short, the ML algorithms have the potential to learn from current context and generalize into unseen tasks and thus could allow improvements in both the safety and efficacy towards better outcomes (Badillo et al., 2020; Choi et al., 2020; Del Rosario & Del Rosario, 2022; Greener et al., 2022; Mjolsness & DeCoste, 2001; Nichols et al., 2019; Zhou et al., 2015). Deep neural network (DNN) also known as deep nets, is a neural network with a certain level of complexity and can be considered as stacked neural networks or the networks that is composed of several layers usually two or more that include input, output and at least one hidden layer in between DDNs and are often employed to deal with unlabeled and unstructured data. They have become the standard tool for solving various computer vision tasks. Briefly, DDNs are among the most widely applied ML and AI tools showing outstanding performance in a broad range of tasks (Kriegeskorte & Golan, 2019; Stelzer et al., 2021).

Recently, the AI‐powered analysis of large‐scale data sets is enabling the identification of patterns and the development of predictive models for early detection and precise diagnosis of medical conditions. In this context, a real‐time monitoring of cardiac function through AI‐driven algorithms using ECG and sonography imaging tools might facilitate an accurate disease management and personalized treatment strategy (Błaziak et al., 2022). Furthermore, integration of blood‐based biomarkers and AI tools can enable the identification of specific molecular signatures associated with HF, leading to targeted interventions, and tailored therapeutic approaches. These advanced AI technologies, coupled with automation, enhance clinical decision‐making, improve patient outcomes, and can contribute to preventive strategies by identifying individuals at high risk for HF and implementing early interventions to mitigate disease progression. Studying the interoception in detail can play a crucial role in mental and cardiovascular health, as mentioned above, because a dysfunctional interoception can have negative consequences that can contribute to cardiovascular complications such as HF. Understanding the intricate interplay between interoception, mental health, and cardiovascular health would serve as the potential key to develop comprehensive strategies for prevention, diagnosis, and treatment of HF integration of advanced technologies encompassing the big data, AI, ML, automation, ECG imaging, sonography, and blood biomarkers (Park et al., 2023). Combined, the newest approaches can offer promising avenues to detect, manage, and prevent HF more quickly. By leveraging these tools, we can enhance clinical decision‐making, improve patient outcomes, and optimize preventive strategies in the pursuit of improved cardiovascular health and well‐being. This review aims to provide insights into the role of interoception in mental health, its consequences for cardiovascular health, and its potential implications for the prevention and management of HF.

## 2 HUMAN BRAIN, INTEROCEPTION, AND THE ROLE OF ARTIFICIAL INTELLIGENCE

**FIGURE 1:**
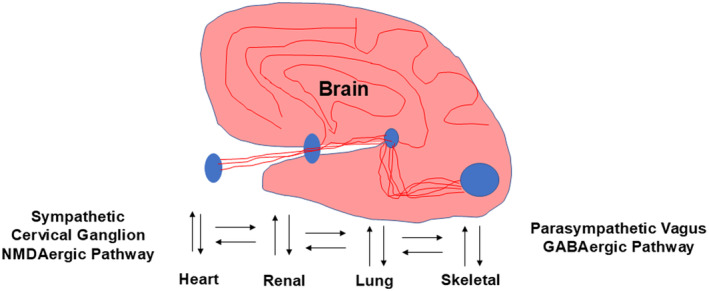
Interoception, Artificial Intelligence (AI), and the human brain. The brain is responsible for interoception, which involves sensing internal bodily signals. Practices like yoga can improve interoception, helping us develop innovative diagnostics and personalized interventions to enhance well‐being and human performance. In this context, advances in AI can certainly provide new research opportunities to deepen our understanding of the interoception.

Interoception, the representation of the body's internal state, plays a central role in emotion, motivation, and well‐being (Zuo et al., 2023). Within the intricate network of the human body, the brain serves as the central command center, orchestrating various physiological processes (Figure 1). One fascinating aspect of our body's intelligence is interoception, the ability to sense and interpret internal bodily signals (Chen et al., 2021). This intricate interplay between mind and body has captivated scientists and led to remarkable advancements in AI. Researchers have recognized the potential of AI in understanding and harnessing the power of interoception for human health and well‐being. Within the ANS, the sympathetic cervical ganglion plays a crucial role in regulating bodily functions through its NMDAergic pathways. Simultaneously, the parasympathetic vagus nerve, with its GABAergic influence, counterbalances sympathetic activity, promoting relaxation and restoration. These intricate neural interactions have a profound impact on various vital organs, including the heart, renal system, lungs, and skeletal system. The heart receives signals from both the sympathetic and parasympathetic divisions, synchronizing its rhythm with the fluctuations in neural activity (Jung et al., 2019). The renal system maintains fluid balance and waste elimination, while the lungs facilitate oxygen exchange to sustain life. The skeletal system provides support and mobility, enabling remarkable feats.

In the pursuit of holistic well‐being, practices like yoga postures and remote ischemia conditioning (RIC) have gained attention. Yoga postures not only promote physical strength and flexibility (Cramer et al., 2013; Field, 2011) but also enhance mental clarity and emotional balance (Gothe et al., 2013; Satyapriya et al., 2013), highlighting the close connection between mind and body. RIC, on the other hand, involves applying brief episodes of ischemia and reperfusion to a limb, triggering protective mechanisms that extend beyond the local tissue (Hausenblas et al., 2015; He et al., 2018). These practices exemplify the complex interplay between various bodily systems and their impact on overall health and well‐being (Bassi et al., 2016; Mason et al., 2014). These practices exemplify the complex interplay between various bodily systems and their impact on overall health and well‐being.

As research advances in understanding brain functions and leveraging AI, we uncover new frontiers in understanding interoception and its profound implications for human health (Edwards & Lowe, 2021). The intricate interplay between the sympathetic and parasympathetic systems offers fertile ground for exploring the potential of AI in deciphering the complexities of interoception. AI algorithms can analyze vast amounts of interoceptive data, identifying patterns and relationships that provide valuable insights into the functioning of the mind–body connection. By integrating AI technologies with our growing understanding of interoception, we can unlock new approaches to promote human health and well‐being. In short, the interplay between interoception, AI, and the human brain represents a fascinating area of exploration. Understanding the intricate connections within the ANS and the effects on vital organs, as well as exploring practices like yoga and RIC, highlights the complex nature of our body's functioning. By utilizing AI to further investigate interoception, we gain a deeper understanding of human health and well‐being (Stern, 2014). This interdisciplinary approach has the potential to revolutionize our understanding and application of interoception, paving the way for innovative strategies to enhance overall health and well‐being.

## 3 DYNAMICS OF THE HEART DURING DIFFERENT PHASES OF ITS CARDIAC CYCLE

**FIGURE 2:**
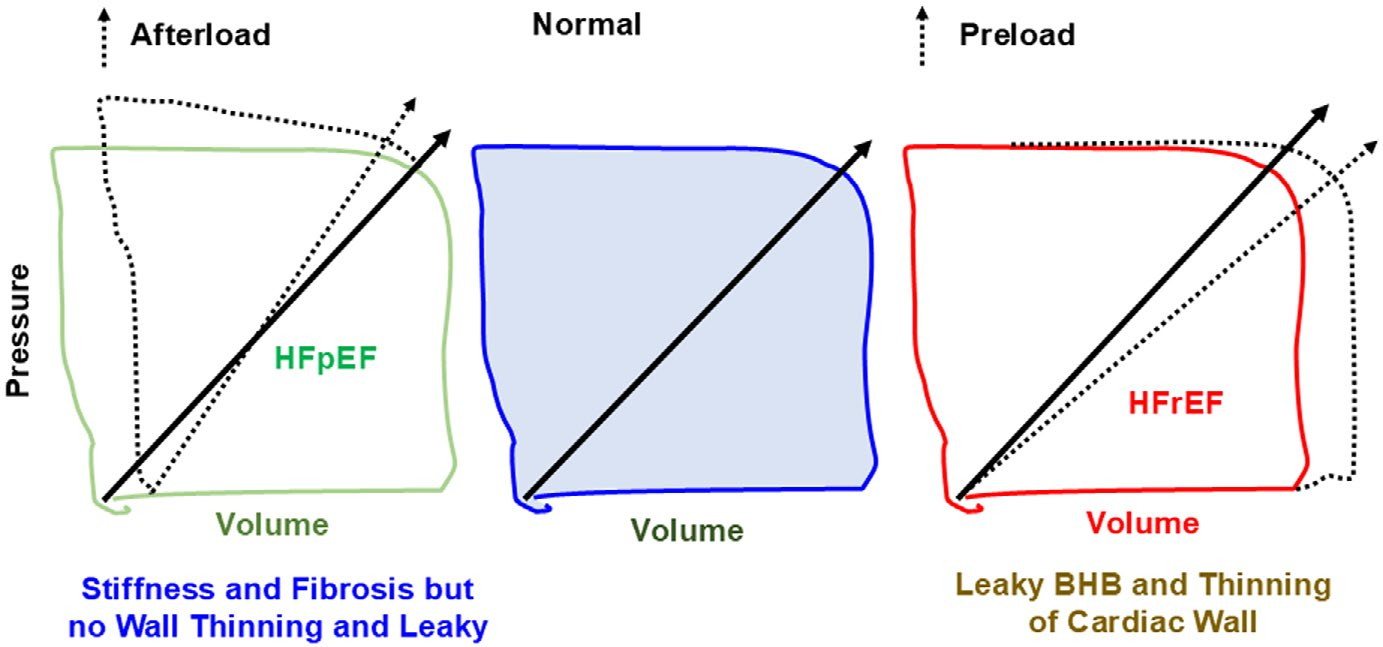
Cardiac Pressure‐Volume Loop. This loop illustrates the dynamics of the heart, showing how it balances preload and afterload for effective pumping. Understanding these variations can help us develop targeted interventions for heart failure (HF), leading to better patient outcomes. BHB refers to the blood‐heart barrier.

The cardiac pressure volume loop offers valuable insights into the heart's function throughout its cardiac cycle (Nakano et al., 2023), illustrating the relationship between cardiac pressure and volume (Figure 2). This understanding can be further enhanced through advancements in AI, ML, automation, big data, imaging, and biomarkers, thus revolutionizing our ability to detect and manage HF effectively. For instance, AI algorithms and ML techniques can analyze large datasets derived from the cardiac pressure volume loop, alongside other patient‐specific information like medical history, genetic data, and biomarker profiles (Averbuch et al., 2022). By scrutinizing these vast datasets, AI can identify patterns and subtle changes in the loop's shape, position, or cardiac parameters, providing early indications of abnormalities or dysfunction in cardiac function. Early detection is crucial for identifying individuals at risk of HF and implementing preventive measures or interventions to manage the condition before it progresses or worsens.

Moreover, the integration of big data in cardiovascular research enables AI algorithms to learn from a diverse range of patient cases, improving the accuracy and efficiency of cardiac imaging analysis. AI‐powered image processing techniques, combined with advanced imaging modalities such as echocardiography, cardiac magnetic resonance imaging (MRI), or computed tomography (CT), can offer detailed assessments of cardiac structure and function (Lim et al., 2020). These technologies facilitate precise quantification of parameters such as ejection fraction (EF), myocardial strain, or total ventricular volumes, enabling early identification of potential abnormalities indicative of HF (Salte et al., 2021). Furthermore, AI‐driven automation can enable real‐time monitoring of cardiac function by continuously analyzing data from wearable devices, such as smartwatches or implantable cardiac devices (Laukkanen & Virtanen, 1998; Prieto‐Avalos et al., 2022). These devices collect a wealth of physiological data, including heart rate, rhythm, and activity levels, which can be easily processed by standardized AI algorithms. By monitoring changes in these data patterns, AI can detect anomalies or deviations from the individual's baseline, providing opportunities for timely interventions and preventive measures to manage HF.

Additionally, the role of AI extends beyond detection to the optimization of treatment strategies for HF. By integrating patient‐specific data, including demographic information, medical history, biomarker profiles, and sequence based genetic data, AI algorithms can assist in tailoring personalized treatment plans for patients. These plans may include selecting appropriate medication regimens, device therapies, and lifestyle interventions, thus helping healthcare providers determine the most effective and efficient treatment options based on individual patient characteristics, risk profiles, and response to previous therapies. Furthermore, the combination of AI technologies with advanced imaging and biomarkers holds promise in improving risk stratification and prognosis assessment for HF. By analyzing complex datasets, AI algorithms can identify novel imaging or biomarker signatures associated with disease progression or treatment response. This knowledge can refine risk prediction models, guide therapeutic decision‐making, and facilitate early intervention in high‐risk individuals. In a nutshell, while the cardiac pressure volume loop serves as a powerful tool in understanding heart dynamics during different phases of its cardiac cycle, integrating AI, ML, automation, big data, imaging, and biomarkers can significantly enhance our ability to understand, detect, prevent, and treat HF. Therefore, leveraging these technologies can enable the analysis of complex cardiac data, identification of early signs of dysfunction, optimization of treatment approaches, and improvement of patient outcomes. We believe that application of AI in cardiac physiology holds promise for personalized medicine and has the potential to revolutionize the management of HF (Johnson et al., 2021).

## 4 THE SIGNIFICANCE OF THE MOLECULAR SIGNATURES, AND APPLICATION OF AI IN HEART FAILURE

**FIGURE 3:**
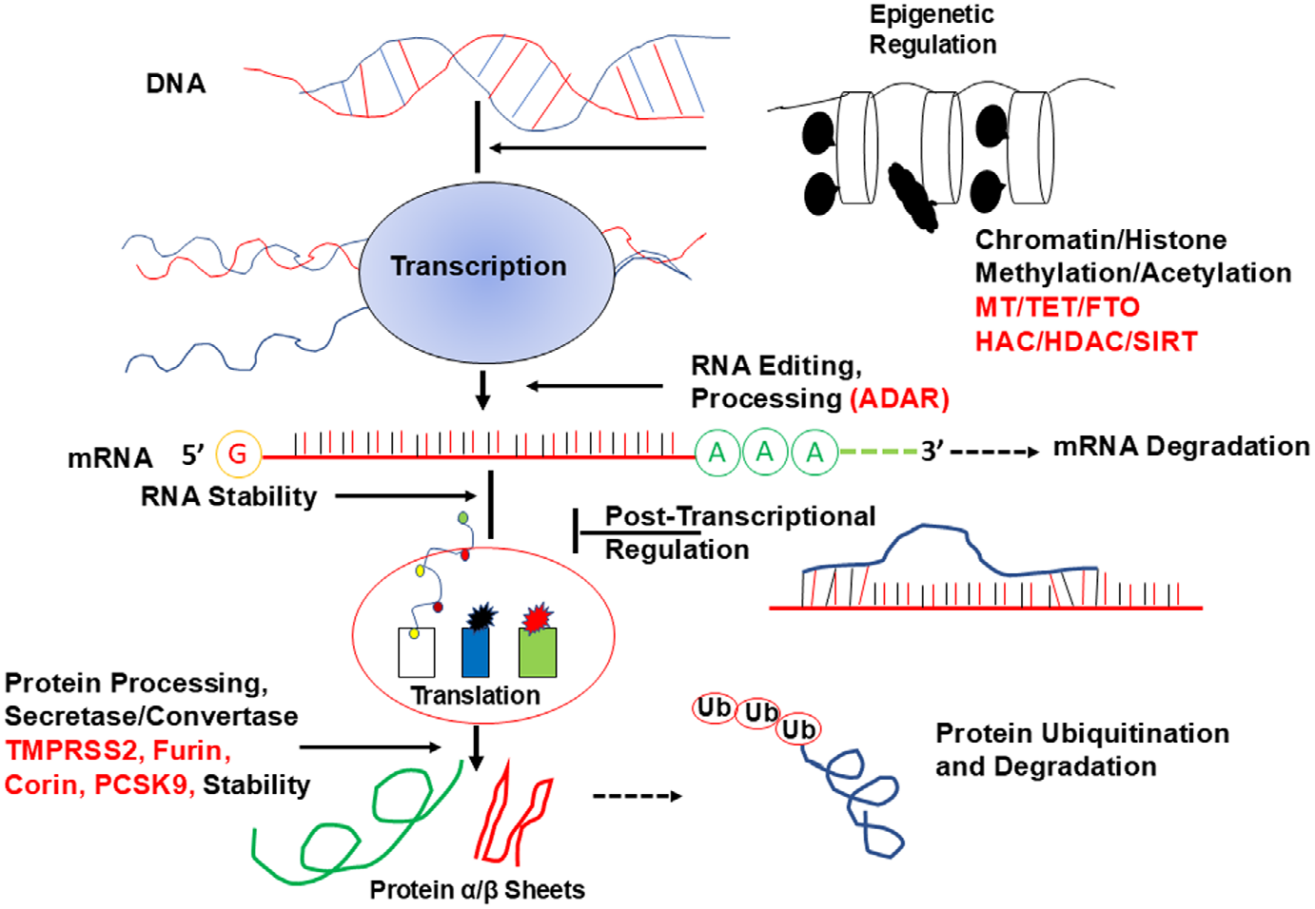
Molecular Footprints in Heart Failure (HF). DNA regulation includes epigenetic processes and transcription control, while proteins and RNA processing affect gene expression and cellular function. Understanding these mechanisms is essential for effectively managing HF. ADAR, adenosine deaminases acting on RNA; FTO, fat mass and obesity‐associated protein; HDAC, histone deacetylase; MT, methyl transferases; PCSK9, proprotein convertase subtilisin/kexin type 9; RNA, ribonucleic acid; SIRT, sirtuin; TET, ten‐eleven translocation proteins; TMPRSS2, transmembrane serine protease 2.

DNA undergoes intricate processes of epigenetic regulation and transcriptional control to modulate gene expression (Rajabian et al., 2023; Tyagi et al., 2021). Chromatin modifications, such as histone methylation and acetylation, play crucial roles in this regulation, ensuring precise gene expression patterns. Enzymes like methyltransferases (MT) and ten‐eleven translocation proteins (TET) dynamically modify DNA, further modulating gene expression (Figure 3) (Arnold & Finley, 2023; Couzin‐Frankel, 2017; Hayden & Tyagi, 2021; Sendžikaitė et al., 2019). Proteins like fat mass and obesity‐associated protein (FTO), HDAC, and SIRT contribute to epigenetic regulation through their involvement in histone modifications and DNA methylation (Mathiyalagan et al., 2019). These mechanisms collectively shape the dynamic control of gene expression and protein synthesis, influencing cellular function. RNA processing and post‐transcriptional regulation are equally vital processes in maintaining proper cellular functioning. For example, RNA editing mediated by adenosine deaminases acting on RNA (ADAR) proteins can alter the coding sequences of RNA molecules, thereby influencing protein synthesis (Kleinova et al., 2023). Post‐transcriptional modifications, including RNA processing, play essential roles in shaping the final functional form of RNA during gene regulation. Furthermore, protein processing involves various enzymes such as secretases, convertases, TMPRSS2, Furin, Corin, and PCSK9, responsible for cleaving, modifying, and activating proteins, ensuring their proper functioning (Lapointe & Couture, n.d.; Dobó et al., 2022; Matveev et al., 2024; Wu & Chen, 2022; Yang et al., 2022). The structural organization of proteins, characterized by α and β sheets, is crucial for their stability and functionality. Additionally, protein ubiquitination and degradation pathways regulate the turnover of proteins, maintaining cellular homeostasis and eliminating damaged or unnecessary proteins.

In the context of HF, the identification of molecular footprints or molecular signatures associated with the condition can be significant. By adopting AI and ML tools, we may improve speed and accuracy in patient diagnosis, such as HF‐related conditions and other serious diseases affecting vital organs (Chow et al., 2024), as summarized in Table 1. We aim to seek the development of an AI algorithm to interpret wide complex tachycardia (WCT) electrocardiograms (ECG) and compare its diagnostic accuracy to cardiologists. By employing advanced AI technologies, including ML, automation, and the analysis of big data, all these tools can play a crucial role in detecting these molecular footprints well in advance in the patient population (Al‐Ani et al., 2023; Averbuch et al., 2022). By leveraging diverse sources of data, including cardiac imaging data, patients' population big data from clinical trials, blood‐based biomarkers, pathological symptoms or signs, family history, lifestyle factors, diets, environmental exposures (such as toxins, chemicals, air pollutants, dust microparticles), infectious diseases, and considering factors like aging, gender, and genetic predisposition, sophisticated AI models or algorithms can be developed to identify patterns and associations that indicate an increased risk of HF (Best et al., 2019). Through careful integration of these diverse datasets and the application of advanced AI models, researchers can uncover molecular signatures that serve as early indicators of HF. These molecular footprints or signatures may encompass genetic variations, epigenetic modifications, altered protein profiles, and specific metabolic or inflammatory markers. By combining information from multiple sources, AI algorithms can identify complex relationships and patterns that are beyond human capacity to discern, facilitating accurate prediction and risk stratification. Moreover, AI technologies can aid in the development of personalized prevention and treatment strategies for HF.

**TABLE 1:** Summation of AI applications in detection of heart failure and other serious medical conditions of vital organs.

S. No.	Medical conditions	Clinical findings/observations	AI‐led outcomes	References
1	Detection of left bundle branch block (LBBB) induced cardiomyopathy and heart failure (HF) arrhythmia	Successful prediction of left bundle branch block‐induced cardiomyopathy and treatment effect AI‐enabled electrocardiogram	AI enabled ECGS helped identify patient who was at risk of developing LBBB‐induced cardiomyopathy and predicted the response to LBBA pacing	(Dhawan et al., 2024)
2	Interpretation of wide complex tachycardia (WCT)	AI appears to diagnose WCT with superior accuracy than Cardiologists and like those of Electrophysiologists	AI algorithm for the interpretation WCT electrocardiograms (ECG) for diagnostic accuracy	(Chow et al., 2024)
3	Cardiac disease detection using an automated Convolutional Neural Network (CNN) system	Successful detection of cardiac arrhythmia such as Arrhythmia (ARR), Congestive Heart Failure (CHF), and Normal Sinus Rhythm (NSR)	A novel approach to cardiac disease detection using an automated CNN system that achieved 99.78% accuracy and an F1 score of 99.78% which is among one of the highest in the models which were recorded to date	(Prusty et al., 2024)
4	Diagnosis and classification of patients with HF	The Atrous Convolutional Neural Network (ACNN) method for HFrEF assessment can avoid issues with human error for diagnosis	The automated neural network‐based calculation of LVEF found to be much faster for evaluations of the cardiac systolic function	(Zhang et al., 2024)
5	Atrial fibrillation during sinus rhythm	Single‐lead ERG AI model can detect atrial fibrillation during sinus rhythm	AI using a single‐lead SR ECG and risk factors can identify concurrent AF with similar accuracy as a 12‐lead ECG‐AI model in an unbiased manner with consistent predictions across age groups	(Dupulthys et al., 2024)
6	Automatic detection of left ventricular hypertrophy	The method can transform waveforms into fixed‐size images and leverage the selected lead of ECG, ensuring adaptability with limited resources. It holds promise enhancing patient outcomes	A robust computationally efficient method that outperforms 1D‐CNN models for LVH detection ensuring adaptability thus holding promise for integration into clinical practice for early diagnosis, earlier treatment, and management	(Cai et al., 2024)
7	HF detection in patients with congenital heart disease (CHD)	A deep‐learning model; Heart failure Attentive Risk Trajectory (hART) for capturing relationships between medical events within patients' history by using mechanism of the past medical events	The hART a deep‐learning model can predict HF trajectories in CHD patients	(Moroz et al., 2024)
8	Assessment for evaluation of AHF	B‐lines assessment by lung ultrasound (LUS) outperform physical exam, chest radiograph, and biomarkers for the associated diagnosis of acute heart failure (AHF) in the emergent setting	The multiclassification AI algorithm is a robust and well performing model at both binary and ordinal multiclass B‐line evaluation for quantitative and objective B‐line assessment for evaluation of AHF	(Pare et al., 2024)
9	Enhancing early detection and diagnosis of HF with non‐invasive monitoring	An integrated approach, combining PPG and ECG signals, demonstrating superior performance compared to single‐signal strategies, emphasizing its potential in early and precise HF diagnosis	Non‐Invasive HF Evaluation Using Machine Learning (ML) Algorithms	(Victor et al., 2024)
10	Identification of left ventricular systolic dysfunction (LVSD)	In patients without LVSD at baseline and follow up, model‐generated high probability for LVSD was associated with a four‐fold increased risk of developing LVSD during follow up	AI‐based ECG‐analyzing model for the detection of LVSD with robust performance metrics	(König et al., 2024)
11	Differentiation of Constrictive Pericarditis (CP) and Restrictive Cardiomyopathy	With a standard apical 4‐chamber view, the AI model facilitated the detection of CP allowing for improved efficiency	AI model to facilitate the detection of CP for advanced evaluation and intervention	(Cau et al., 2024; Chao et al., 2024)
12	The diagnosis of cardiac amyloidosis	The medical algorithmic audit demonstrated AI robustness across demographic factors, tracers, scanners, and centers. AI's predictions were independently prognostic for overall mortality	AI‐based screening of cardiac amyloidosis is reliable and can eliminate inter‐rater variability, and portended prognostic value, with potential implications for identification, referral, and management	(Spielvogel et al., 2024)
13	Detection of Takotsubo Cardiomyopathy (TC)	This approach is beneficial for stratifying high‐risk patients with TC by AI‐ECG as a significant predictor of major adverse cardiac events in patients with TC	Combined use of AI‐ECG algorithms detects patterns associated with worse outcomes in TC	(Kanaji et al., 2024)
14	Detection of undiagnosed HF with preserved ejection fraction	The application of natural language processing (NLP) pipeline to electronic health record (EHR) helped identify patients with a clinical diagnosis of HF	AI for improved detection of undiagnosed HF using NLP can identify likely HFpEF patients from EHR data who would benefit from expert clinical review and complement the use of diagnostic algorithms	(Oo et al., 2024; Wu et al., 2024)
15	Detection of peripartum cardiomyopathy	AI‐based HF detection model for peripartum cardiomyopathy detection using only lead‐I electrocardiograms led to high detection accuracies	AI as a noninvasive tool for detecting peripartum cardiomyopathy using electrocardiogram data	(Karabayir et al., 2024)
16	Determination of mental health, interoception, psychological flexibility through linear associations alexithymia as an outcome	Higher levels of alexithymia were associated with increased psychological inflexibility, lower positive affect scores, and lower interoception	Self‐as‐Context (SAC) processed‐based therapeutic model for alexithymia	(Edwards & Lowe, 2021)
17	Decision for the critically brain injured patients via AI and ML	Advancement in computational capabilities for implementing ML in clinical settings, allowing for real‐time analysis and decision support at the point of care (POC)	AI and ML applications in critically brain injured patients	(Vitt & Mainali, 2024)
18	Predicting the occurrence and progression of Alzheimer's Disease (AD)	MRI‐based hippocampal volume measurement with AI can help diagnose and intervene in AD progression	AI deep learning technology for early intervention and prevention of AD	(Zhou et al., 2024)
19	Detection of Hydronephrosis and Precise Kidney Segmentation	Hydronephrosis is crucial in the diagnosis of renal colic and AI allowed automated detection of hydronephrosis with high accuracy	Automated ultrasound deep learning model can be used for the diagnosis of acute renal failure	(Alexa et al., 2024)
20	Diagnosis and prognosis of sight‐threatening eye diseases and prediction of HF and myocardial infarction	RETFound outperformed comparison models in the diagnosis and prognosis of eye diseases and complex systemic disorders from retinal imaging	Medical AI RETFound model uses retinal images for disease detection	(Zhou et al., 2023)

By analyzing the intricate interactions between genetic factors, environmental exposures, lifestyle choices, and molecular footprints, AI algorithms can generate tailored interventions aimed at mitigating the risk and progression of HF (Leiner et al., 2019). This includes optimizing medication regimens, implementing lifestyle modifications, and identifying novel therapeutic targets based on an individual's unique molecular profile and disease trajectory. Hence, the molecular footprints in HF reflect the intricate molecular mechanisms underlying gene expression, protein synthesis, and cellular function. Together, AI, ML, automation, and big data analysis can play a crucial role in detecting these molecular underpinnings and help develop predictive models for early detection, prevention, and treatment (Martin‐Isla et al., 2020). In brief, by unraveling the complex interactions between genetics, epigenetics, environmental factors, lifestyle choices, and molecular footprints, AI technologies can pave the way for personalized approaches to address the multifaceted nature of HF and that might prove to be highly useful in improving patient outcomes (Venkat et al., 2023).

## 5 THE POTENTIAL IMPLICATIONS OF EPIGENETICS IN HEART FAILURE, AND INTEGRATION OF THE AI KNOWLEDGE

**FIGURE 4:**
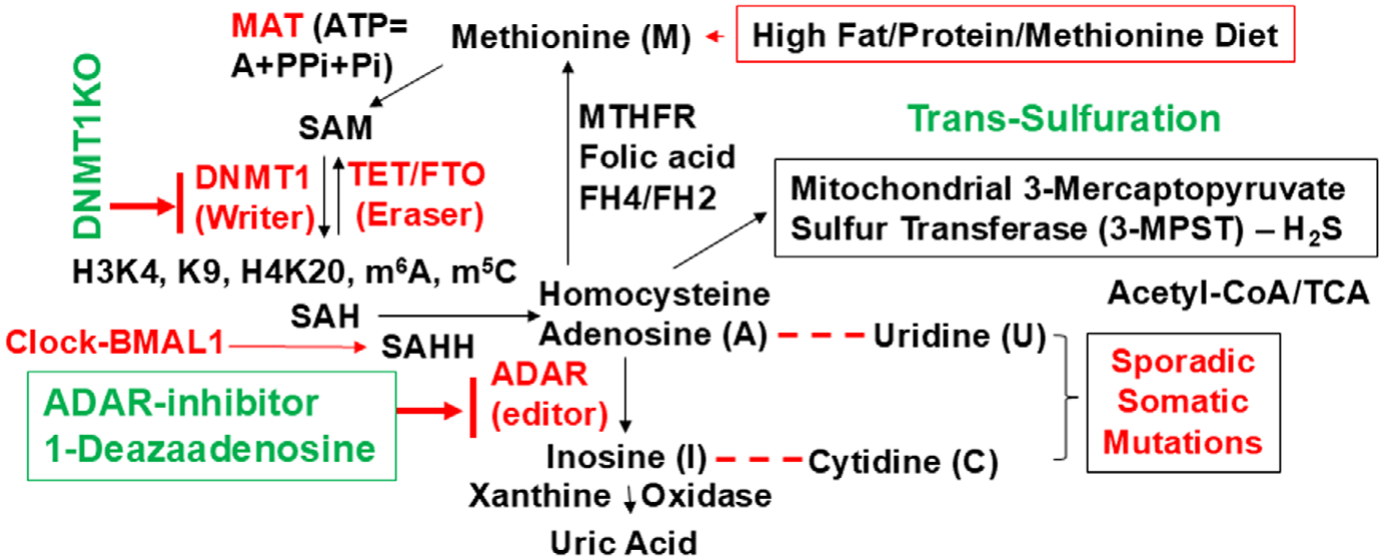
Dysrhythmic Gene Modifiers in HFrEF. Heart failure (HF) with reduced ejection fraction (HFrEF) is linked to dysregulated gene expression and epigenetic changes. Key factors and metabolic processes influence these epigenetic modifications. Understanding these elements is vital for developing effective interventions. DNMT, DNA methyltransferase, FTO, fat mass and obesity‐associated protein; MTHFR, methylenetetrahydrofolate reductase; TET, ten‐eleven translocation proteins.

In heart failure with a reduced ejection fraction (HFrEF), dysregulation of gene expression plays a critical role in pathophysiology. Dysrhythmic gene writers, erasers, and editors are key players that control epigenetic modifications in cardiac tissue, contributing to the molecular landscape of HFrEF (Krittanawong et al., 2022). A recent clinical trial has shown improvement in HFrEF by increasing myosin binding protein and factors that phosphorylate it (Teerlink et al., 2021; Teerlink, Diaz, Felker, McMurray, Metra, Solomon, Adams, et al., 2020; Teerlink, Diaz, Felker, McMurray, Metra, Solomon, Legg, et al., 2020). These molecular players include enzymes and proteins involved in the regulation of DNA methylation, histone modifications, RNA processing, and protein modifications. One important molecular player is the methionine adenosyl transferase (MAT) enzyme, responsible for synthesizing S‐adenosylmethionine (SAM), a crucial methyl donor in DNA methylation. SAM is involved in the addition of methyl groups to DNA, regulating gene expression patterns (Rajabian et al., 2023). Dysregulation of MAT activity due to factors such as a high‐fat diet, high‐protein diet, or high methionine diet can influence the availability of SAM and impact gene expression patterns in HFrEF. Similarly, the dysregulation of other enzymes and proteins involved in DNA methylation dynamics, such as the ten‐eleven translocation (TET) proteins, fat mass and obesity‐associated (FTO) protein, and methylenetetrahydrofolate reductase (MTHFR) enzyme, can disrupt the epigenetic landscape in HFrEF. As we know that the TET proteins are responsible for DNA demethylation processes, and therefore, alterations in their activities can impact gene expression patterns. FTO protein and MTHFR enzyme are involved in the metabolism of key molecules associated with epigenetic modifications, such as m6A (N6‐methyladenosine) and methyl donors like folic acid. Dysregulation of FTO and MTHFR can impact the availability of these molecules, thereby affecting gene expression and epigenetic regulation in HFrEF (Figure 4).

Furthermore, disturbances in the trans‐sulfuration pathway, involving the enzyme mitochondrial 3‐mercaptopyruvate sulfur transferase (3‐MST) and the production of hydrogen sulfide (H2S), can influence DNA methylation patterns and histone modifications in HFrEF. Trans‐sulfuration pathway disruptions can alter H2S levels, which act as signaling molecules with potential cardioprotective effects. Changes in H2S levels can impact the epigenetic modifications associated with gene expression regulation. Apart from DNA methylation and histone modifications, dysregulation in RNA processing and post‐transcriptional regulation can also contribute to the molecular landscape of HFrEF. As mentioned earlier, enzymes like RNA editing mediated by adenosine deaminases acting on RNA (ADAR) proteins can introduce modifications in RNA molecules, altering their coding sequences and influencing protein synthesis. Additionally, post‐transcriptional modifications shape the functional form of RNA and involve processes such as RNA editing and alternative splicing. So, a dysregulation in these processes can impact gene expression patterns and contribute to the development and progression of HFrEF. it should be noted that metabolic imbalances in HFrEF patients, including elevated levels of xanthine oxidase and uric acid, can promote oxidative stress and inflammation, further impacting gene expression and epigenetic regulation (Doehner et al., 2022). Also, dysregulation of molecules such as SGLT2, NGAL, FGF23, TMPRSS2, and MMP‐2, ‐9, ‐13 can contribute to cardiac remodeling and dysfunction as often observed in HFrEF (Yang et al., 2021). Hence, understanding the intricate interplay between dysrhythmic gene writers, erasers, and editors, along with diet, metabolic factors, and circadian rhythms, is crucial for unraveling the epigenetic mechanisms underlying HFrEF. This knowledge can pave the way for the development of novel therapeutic strategies that target epigenetic modifications to mitigate the progression of the disease and improve patient outcomes (Rauschert et al., 2020).

Further, in the context of HFrEF, advanced AI technologies can assist in detecting the HF phenotype, identifying dysregulated gene expression patterns, and facilitating personalized prevention and treatment strategies. By integrating diverse datasets, including imaging data, patients' population big data, and biomarkers, AI models and algorithms can identify patterns and associations that indicate the malfunctioning of the heart leading to HF. These sophisticated AI technologies can enable the analysis of complex interactions between genetic factors, environmental exposures, lifestyle choices, and epigenetic modifications to provide insights into the development and progression of HFrEF. By leveraging big data analysis and AI algorithms, researchers can identify the dysrhythmic gene writers, erasers, and editors associated with HFrEF and their impact on gene expression and epigenetic regulation. Furthermore, AI‐based predictive models can aid in the early detection of HFrEF and the identification of individuals at high risk, allowing for timely interventions and preventive strategies. Additionally, AI‐driven approaches can assist in optimizing treatment strategies by considering individual patient characteristics, such as genetic profiles, epigenetic modifications, and environmental factors, to develop tailored therapeutic interventions. We opine that an integration of AI technologies into the management of HFrEF holds great potential for improving patient outcomes and revolutionizing the field of cardiovascular medicine.

## 6 CONCLUSIONS

Interoception is a process by which our central nervous system (CNS) senses, interprets, and integrates signals originating from within the body. As nicely defined by Sherrington, interoception refers to the “stimuli that originate inside the body,” and comprises all sensations coming from the gastrointestinal, urinary, or reproductive tracts, circulatory or respiratory systems (Sherrington, 1906). In other words, it is the body‐to‐brain axis of sensation concerning the state of the internal body and its visceral organs (Cameron, 2001). This helps provide a “moment‐by‐moment” map of our body's internal landscape across the two separate worlds of consciousness and unconsciousness. In other words, interoceptive signaling has been considered a process of feelings, reflexes, drives, urges, adaptive responses, and cognitive and emotional experiences, thus highlighting its role in the maintenance of body regulation, that is, homeostatic functioning, and ultimately survival. Any interference in the functioning of interoception can lead to dysfunction of our mental health that has a direct effect on body systems. The previous concepts of interoception tended to focus on visceral sensations only, but current concepts describe interoception as a sense of the physiological condition of the body that essentially includes a much wider range of physiological sensations, including muscular effort or vasomotor sensations. These sensations are triggered by stimulation of free nerve endings (the unmyelinated sensory nerve endings) that project to the insular cortex rather than to the primary somatosensory cortex. Feelings arising from these sensations are not sensorial, but they also have an affective and motivational aspect that relates to the homeostatic needs of our body. They are supposed to be associated with our behavioral motivations and are essential for maintaining our physiological integrity (Berlucchi & Aglioti, 2010).

In this work, we explored the intriguing intersection of AI, interoception, cardiac health, and HF. Our investigation aimed to shed light on the potential of AI technologies to enhance our understanding, assessment, and management of cardiac conditions, particularly HF. The findings presented here demonstrate the promising role of AI in revolutionizing the field of cardiovascular medicine and improving patient outcomes. Interoception, the perception, and awareness of internal bodily states, play a vital role in cardiac health and are closely linked to HF. By harnessing AI algorithms, we have witnessed remarkable advancements in the interpretation and analysis of interoceptive signals, enabling us to gain deeper insights into the complex interplay between physiological and psychological factors that impact cardiac function. AI techniques, such as ML and deep neural networks (DNN), have shown remarkable proficiency in detecting subtle changes in interoceptive signals and identifying patterns indicative of HF risk (Mohammad et al., 2022). One of the key contributions of this review lies in the development of AI‐based predictive models for HF risk assessment. By leveraging large‐scale datasets, incorporating various interoceptive measurements, clinical parameters, and demographic factors, these models exhibit significant potential in identifying individuals at high risk of developing HF. This early identification can facilitate timely interventions, leading to better management strategies and improved patient outcomes. Furthermore, AI has also demonstrated its efficacy in enhancing diagnostic accuracy and precision. By integrating AI algorithms into diagnostic processes, clinicians can access real‐time, data‐driven insights, aiding in the accurate identification and differentiation of HF subtypes. The ability to leverage AI‐powered tools, such as image recognition algorithms for cardiac imaging analysis, has shown immense promise in improving the accuracy of diagnoses, reducing the likelihood of misclassifications, and guiding appropriate treatment strategies. It is important to note that while AI holds great promise in the realm of cardiac health, its successful integration into clinical practice necessitates thoughtful considerations. Addressing issues related to data privacy, algorithm transparency, and ethical concerns is crucial for building trust and ensuring the responsible implementation of AI technologies in healthcare settings.

**FIGURE 5:**
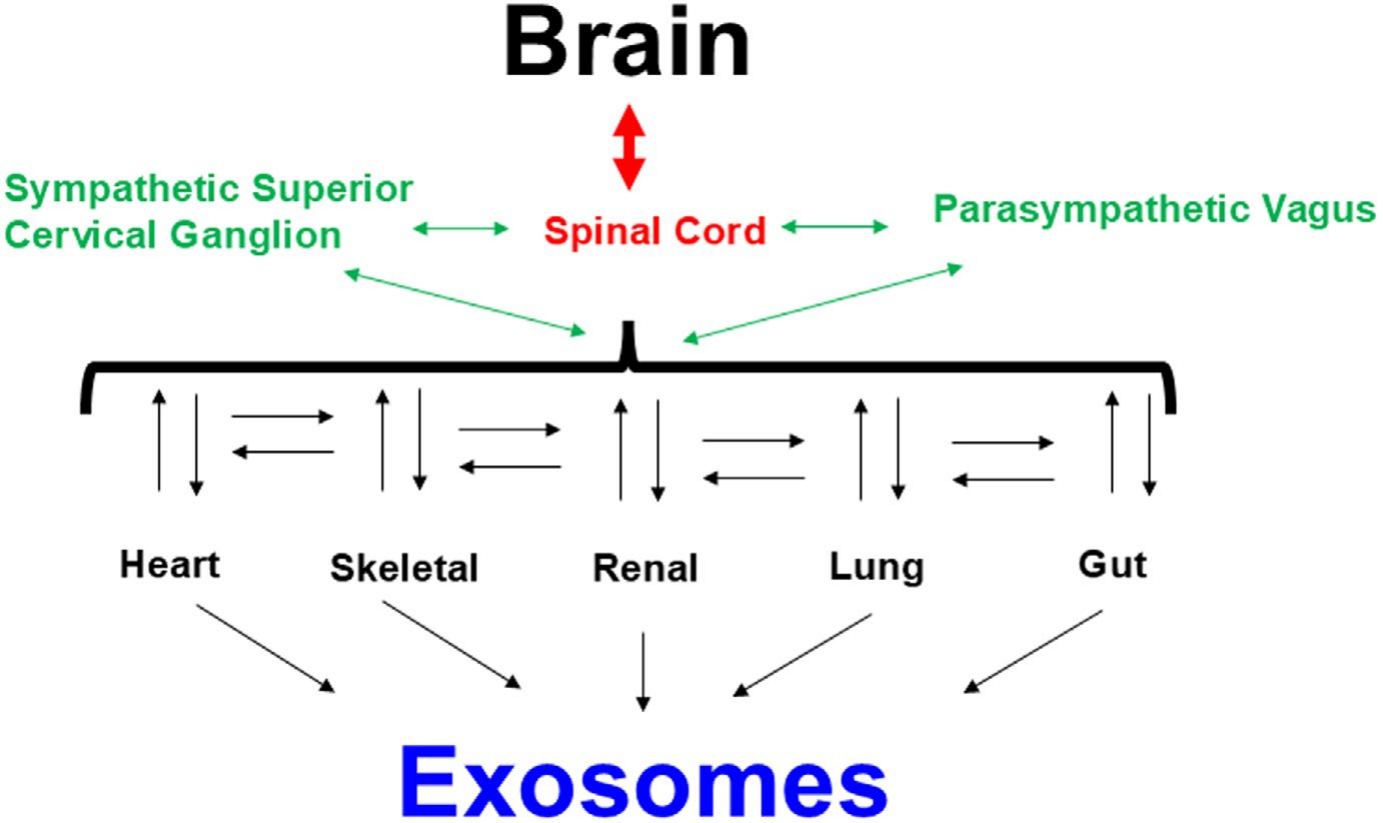
Interoception and AI. Schematic of major inter‐organ communication networks of the body (please see the text for details).

Recent experimental evidence in patients with disorders of consciousness has shown that observing brain‐heart interactions can help detect residual consciousness, even in patients without behavioral signs of consciousness (Candia‐Rivera, 2022). The analogy mentioned earlier, “I see, I forget, I read, I remember, I read by speaking, I do remember, and I read by writing/doing (i.e., akin to ML) and I do not forget, i.e., interoception,” can be explained by the way the brain communicates with systems, and how systems communicate with each other during stress and happiness, that is, interoception. This extends to a similar intelligent network, which is the exciting field of AI progressing in many domains of human activities. When considering meditation and yogic practices, where the mind is at rest, but the posture moves the organs, it sends parasympathetic signals to different organs via communication channels such as exosomes (Figure 5). Furthermore, during remote ischemic conditions, the mind sends parasympathetic signals that increase hemodynamic changes in other organs. Similarly, physical exercise sends information through sympathetic and parasympathetic signals, influencing the hemodynamic environment of other organs. Although interoception is linked to the brain communicating with other organs, a few studies have shown that during deep sleep (when the brain is resting), leg movements cause changes in heart rate, blood pressure, and heart rate variability. This suggests that the skeletal system communicates with the heart's functioning. This implies that, in addition to the brain, there is communication between systemic organs (Malkiewicz et al., 2024). Recently, Singh M and colleagues made an interesting observation that during HF, the interoceptive process via exosomes might be involved in transitioning from HFpEF to HFrEF phenotypes (2nd Annual National Institutes of Health; NIH, Investigators' Meeting on Interoception Research, November 11, 2023, NIH campus, Hyatt Regency Bethesda, Maryland, Washington, D.C.; USA).

AI has the potential to transform how we diagnose, develop biomarkers, and treat HF. It can analyze vast amounts of patient data like images, genetic information, and medical records to detect HF early and classify its types accurately. However, there are challenges, particularly regarding the complexity of AI algorithms, which may make it difficult to understand the reasoning behind diagnostic decisions, leading to concerns about trust and accountability in clinical practice. In biomarker development, AI can identify new molecular and imaging markers for predicting HF risk and prognosis by uncovering hidden patterns in diverse datasets. But the reliability and applicability of AI‐derived biomarkers are under scrutiny due to variations in data quality and model performance across different patient groups. Similarly, in treatment, AI‐powered decision support systems can assist in personalized therapy selection, dosage adjustment, and device management based on individual patient characteristics. However, integrating AI into clinical workflows requires rigorous validation, regulatory compliance, and ethical considerations to ensure patient safety, data privacy, and equitable access to AI‐enabled healthcare solutions. Further, technical challenges such as data bias, algorithmic transparency, and model interpretability pose significant hurdles to the widespread adoption of AI in HF care. Addressing these challenges requires collaboration among clinicians, data scientists, ethicists, and regulatory agencies to develop transparent, accountable, and clinically validated AI solutions that complement human expertise in cardiovascular medicine. Furthermore, ethical concerns are also paramount in AI‐driven healthcare. Safeguarding patient data privacy, mitigating algorithmic bias, and preserving human agency in decision‐making are essential ethical imperatives that require interdisciplinary cooperation and regulatory oversight to ensure fairness, safety, and respect for patient autonomy in AI‐enabled healthcare.

Regarding the future direction in advancing AI‐driven HF management, we opine that future research should focus on enhancing the interpretability of AI models to provide clinicians with actionable insights into diagnostic and treatment decisions. Large‐scale multicenter studies are needed to validate the reliability and generalizability of AI‐derived biomarkers across diverse patient populations, addressing variability in data quality and harmonizing data collection protocols. Prospective clinical trials and implementation studies are essential to evaluate the real‐world clinical impact of AI‐driven decision support systems on patient outcomes, workflow integration, and clinician acceptance. Furthermore, ethical guidelines and regulatory frameworks should be developed to govern the responsible use of AI in HF care, ensuring equity, transparency, and accountability in the development and deployment of AI‐enabled healthcare solutions. Again, interdisciplinary collaboration between clinicians, data scientists, ethicists, and policymakers are crucial for translating AI research findings into clinical practice and enhancing healthcare professionals' capacity to critically evaluate and utilize AI‐driven interventions in HF management. Through these efforts, the field of AI‐driven HF management can advance towards the development of more effective, equitable, and patient‐centered healthcare solutions, ultimately improving outcomes for individuals living with HF.

In conclusion, this review highlights the transformative potential of AI in advancing our understanding of interoception, enhancing cardiac health assessment, and optimizing HF management based on existing research. (Schepart et al., 2023). By leveraging AI algorithms, we can uncover hidden patterns within interoceptive signals, improve risk prediction, and enhance diagnostic accuracy. These advancements have the potential to revolutionize cardiac care, leading to personalized treatment approaches, improved patient outcomes, and ultimately a reduction in the burden of HF. However, careful attention must be given to ethical considerations and regulatory frameworks to ensure the responsible and equitable use of AI in healthcare. Future research endeavors should focus on refining AI algorithms, validating their performance in diverse populations, and addressing implementation challenges to fully unlock the transformative potential of AI in the field of cardiovascular medicine (Canning et al., 2023).

## AUTHOR CONTRIBUTIONS

Mahavir Singh and Suresh C. Tyagi conceived the idea of the manuscript and wrote the manuscript. Anmol Babbarwal and Sathnur Pushpakumar edited and helped in collecting the material for the manuscript.

## FUNDING INFORMATION

The authors express special thanks to the funding sources (NIH: HL‐74185, HL‐139047, DK116591 and AR‐71789).

## CONFLICT OF INTEREST STATEMENT

The authors declare that they have no conflict of interest, financial or otherwise.

## ETHICS STATEMENT

N/A.

## CONSENT TO PARTICIPATE

N/A.

## Data Availability

N/A.
